# Pomiferin Exerts Antineuroinflammatory Effects through Activating Akt/Nrf2 Pathway and Inhibiting NF-*κ*B Pathway

**DOI:** 10.1155/2022/5824657

**Published:** 2022-04-04

**Authors:** Yan Zhao, Yuxuan Sang, Yanan Sun, Jie Wu

**Affiliations:** ^1^Department of Radiation Oncology & Therapy, The First Hospital of Jilin University, Changchun 130021, China; ^2^Jilin Provincial Key Laboratory of Radiation Oncology & Therapy, The First Hospital of Jilin University, Changchun 130021, China; ^3^NHC Key Laboratory of Radiobiology, School of Public Health, Jilin University, Changchun 130021, China; ^4^Harbin Medical University (Daqing), College of Medical Laboratory and Technology, China

## Abstract

**Background:**

Neurodegenerative diseases, including Alzheimer's disease, Parkinson's disease, and Huntington's disease, are mainly characterized by progressive motor, sensory, or cognitive dysfunction in patients. Such diseases mostly occur in middle-aged and elderly people, and there is no effective cure. Studies have shown that neurodegenerative diseases are accompanied by neuroinflammation. The proinflammatory mediators produced neuroinflammation further damage neurons and aggravate the process of neurodegenerative diseases. Therefore, inhibiting neuroinflammation might be an effective way to alleviate neurodegenerative diseases. Pomiferin extracted from the fruit of the orange mulberry has a wide range of antioxidation and anti-inflammatory effects in peripheral tissues. However, it is not clear whether it plays a role on neuroinflammation.

**Methods:**

In our experiment, we studied the effect of Pomiferin on BV2 cell inflammation and its mechanism with cck-8, LDH, quantitative PCR, and ELISA and methods. We then investigated the effect of Pomiferin on the classical inflammatory pathway by Western blot methods.

**Results:**

The results showed that Pomiferin inhibited the production of ROS, NO, and proinflammatory mediators (IL-6, TNF-*α*, iNOS, and COX2) in BV2 cells. Further mechanism studies showed that Pomiferin activated the Akt/Nrf2 pathway and inhibited the NF-*κ*B pathway.

**Conclusion:**

Our study demonstrated that Pomiferin exerts antineuroinflammatory effects through activating Akt/Nrf2 pathway and inhibiting NF-*κ*B pathway.

## 1. Introduction

Degenerative diseases are diseases in which the structure or function of neurons is gradually lost, including death, leading to dysfunction, such as Parkinson's disease (PD), Alzheimer's disease (Alzheimer's disease), and Huntington's disease (HD). These diseases are mainly characterized by neurodegeneration in one or more parts of the central nervous system, with a long course and complex pathogenesis [1–3]. At present, it can only be alleviated in the early stage clinically, and there is no effective cure. Therefore, it is of great significance to study the pathogenesis of neurodegenerative diseases and search for effective therapeutic drugs.

Neuroinflammation is an immune response activated by microglia and astrocytes in the central nervous system (CNS). Studies have shown that microglia are activated by multiple factors, such as CNS injury, brain infection, toxin stimulation, and autoimmune diseases. Autopsy reports indicate that the brains of patients with neurodegenerative diseases have large aggregates of microglia [4–6]. Studies also have shown that neuroimmunity and inflammatory responses are involved in the process of neurodegenerative diseases [7–9]. In the event of disease, immune cells are activated in large numbers and flock to the site of injury to remove harmful factors and repair damaged tissue. However, persistent inflammatory response leads to excessive activation of local immune cells in the central nervous system and accumulation of large amounts of toxic mediators such as IL-6, TNF-*α*, NO, and PEG2, which further damages peripheral neurons and aggravates the progression of neurodegenerative disease [10, 11]. The dysfunction of neuronal cells in specific regions caused by oxidative stress is an important pathogenesis of neurodegenerative diseases, in which the excessive production of ROS is an important pathology [12]. Therefore, it is of great significance to suppress the overactivation of immune cells and the neuroinflammatory response caused by it for the remission of neurogenic diseases.

Pomiferin is a kind of flavonoids extracted from the fruit of the orange mulberry. Studies show that Pomiferin has antioxidation and anti-inflammatory effects. In the oxidative damage model, Pomiferin has a protective effect on the DNA damage caused by catalpa glucoside, showing a strong antioxidant activity [13, 14]. In macrophages, Pomiferin activates the nuclear factor infrared 2-related factor 2 (Nrf2), triggers the transcription of antioxidant genes, exerts the antioxidant effect and the degradation of I*κ*B *α*, and plays an anti-inflammatory role [15, 16]. In human skin fibroblasts (NHDF) and normal human skin keratinocytes, Pomiferin can upregulate antioxidant genes such as ACLY, AQP3, and COX1 [17, 18]. Given its extensive antioxidant and anti-inflammatory activity, we hypothesized that Pomiferin might have an effect on neuroinflammation. Therefore, the purpose of this study was to explore the effect and mechanism of Pomiferin on neuroinflammation.

## 2. Materials and Methods

### 2.1. Reagent

LPS (from Escherichia coli, O55:B55) was obtained from Sigma (Aldrich, St. Louis, MO, USA). Fetal bovine serum (FBS), trypsin, and DMEM high-glucose medium were purchased from (Gibco, Grand Island, NY, USA). Penicillin and streptomycin were purchased from Invitrogen (Carlsbad, CA, USA). Pomiferin (>98%purity; Pufei De Biotech, Chengdu, China) was dissolved in DMSO (Sigma Aldrich St Louis, MO, USA). MK2206 (an Akt inhibitor) and brusatol (BT, a Nrf2 inhibitor) were purchased from Selleck (Shanghai Blue Wood Chemical Co., Ltd., China). ROS, LDH, NO detection kit, and CCK-8 reagent were purchased from Beyotime Biotechnology (Shanghai, China).

### 2.2. Cell Culture

We incubated BV2 cells (purchased from Shanghai Binsui Biological (Shanghai, China)) in complete medium (high glucose, 10% FBS, 100 U/mL penicillin, 100 *μ*g/mL streptomycin) in a 5% carbon dioxide cell incubator at 37°C. The cell culture medium is replaced daily. When the density reached about 80%, the cells were digested with 0.05% trypsin and subcultured into new cell culture flask.

### 2.3. CCK-8 Assay

When the density of BV2 cells inoculated in 96-well plates reached about 50%, the cells were treated with different concentrations of Pomiferin (0.25 *μ*M, 0.5 *μ*M, and 1 *μ*M) and LPS (500 ng/mL) for 24 h. Then, we added CCK-8 diluent to each well and incubated it in a cell incubator for 2 h. After that, the survival rate of the cells was measured at the absorbance of 450 nm.

### 2.4. LDH Assay

When the density of BV2 cells inoculated in 96-well plates reached about 80%, the complete culture medium was changed to incomplete culture medium. Then, the cells were treated with different concentrations of Pomiferin (0.25 *μ*M, 0.5 *μ*M, and 1 *μ*M) and LPS (500 ng/mL) for 24 h. After that, according to the instructions of the LDH detection kit, the LDH working fluid was added to the 96-well plates, and the release of LDH was detected according to the LDH manufacturer's instructions.

### 2.5. ROS Measurement

When the density of BV2 cells inoculated in 96-well plates reached about 80%, we added Pomiferin (1 *μ*M) and LPS (500 ng/mL) to treat the cells for 12 h. Then, the ROS level was measured with a ROS detection kit according to the manufacturer's instructions.

### 2.6. NO Measurement

When the density of BV2 cells inoculated in 96-well plates reached about 80%, we added Pomiferin (1 *μ*M) and LPS (500 ng/mL) to treat the cells for 12 h and obtained the cell supernatant. Then, the concentration of NO in the supernatant was measured using a NO detection kit according to the NO manufacturer's instructions.

### 2.7. Quantitative PCR

When the density of BV2 cells inoculated in 96-well plates reached about 80%, the complete culture medium was changed to incomplete culture medium. We added Pomiferin (1 *μ*M) and LPS (100 ng/mL) to treat the cells for 12 h and extracted RNA using the Trizol reagent (Sigma-Aldrich, St. Louis, MO, USA). RNA is reverse-transcribed into cDNA using the Prime Script®1stStrand cDNA Synthesis Kit (Roche, South San Francisco, CA, USA). After that, the mRNA level of IL-6, TNF-*α*, iNOS, and COX-2 were measured with Quanti Tect SYBR Green RT-PCR Kit (Takara Biotechnology, Ltd., Kyoto, Japan) according to the ELISA manufacturer's instructions. Primer sequences refer to previous studies [19, 20].

### 2.8. ELISA

When the density of BV2 cells inoculated in 96-well plates reached about 80%, the complete culture medium was changed to incomplete culture medium. We added Pomiferin (1 *μ*M) and LPS (500 ng/mL) to treat the cells for 24 h, and obtained the cell supernatant. The protein concentration of IL-6 and TNF-*α* in the supernatant was measured according to the ELISA manufacturer's instructions.

### 2.9. Western Blot

Total cell proteins were extracted using a protein extraction kit according to the manufacturer's instructions (Beyotime Biotechnology, Shanghai, China). A total of 50 *μ*g of protein was added to the well for electrophoresis. After electrophoresis, the proteins were transferred to fibrous PVDF membranes (Millipore, Billerica, MA, USA). Then, the membrane was placed in 5% skim milk powder and sealed for 4 h. After that, the membrane was incubated with the primary antibody at 4°C for 12 h and the secondary antibody at room temperature for 2 h. After cleaning with a membrane wash solution, the membrane is colorized with an ECL luminescent solution, and the proteins are imaged with a protein imager. All primary antibodies are purchased from Abcam (Cambridge, UK) and the secondary antibody is purchased from Biological Technology (Wuhan, China).

### 2.10. Data Analysis

All data were presented in mean ± SD form and analyzed using SPSS 19.1 software. One-way analysis of variance was used to compare the different groups. *P* values < 0.05 were considered statistically significant.

## 3. Results

### 3.1. Effect of Pomiferin on the Growth of BV2 Cells

**Figure 1:**
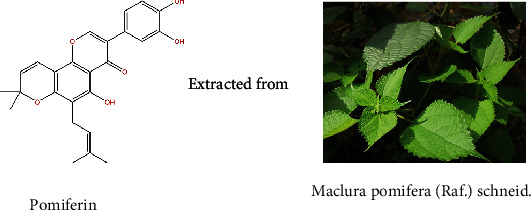
(a) The chemical formula of Pomiferin. Pomiferin was extracted from Maclura pomifera (Raf.) Schneid.

Pomiferin is a kind of flavonoids extracted from Maclura pomifera (Raf.) Schneid (Figure 1).

**Figure 2:**
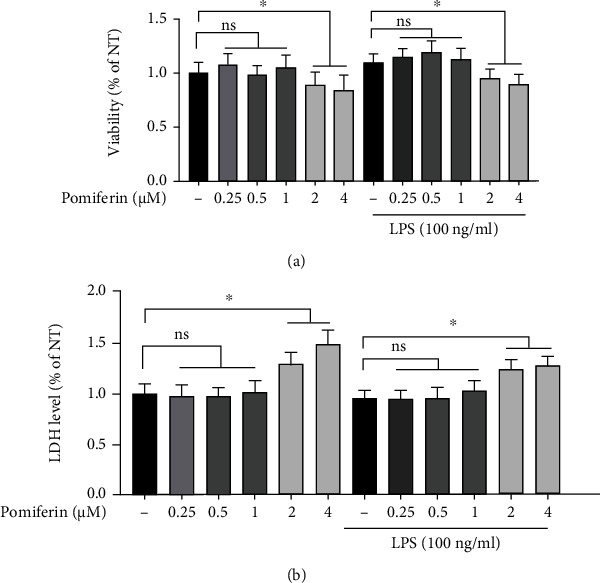
Effect of Pomiferin on the growth of BV2 cells. BV2 cells were treated with LPS (100 ng/mL) and Pomiferin (0.25 *μ*M, 0.5 *μ*M, and 1 *μ*M). (a) The viability of BV2 cells was measured by CCK8 method. (b) The LDH release was detected by LDH experiments. Results were shown via mean ± SD (*n* = 10).

In order to study whether Pomiferin has toxic effects, BV2 cells were treated with LPS (100 ng/mL) and different concentrations of Pomiferin (0.25 *μ*M, 0.5 *μ*M, and 1 *μ*M), and then, the effects of Pomiferin on the growth of BV2 cells were detected by CCK-8 (Figure 2(a)) and LDH (Figure 2(b)) experiments. The results showed that LPS and Pomiferin had no toxic effects on BV2 cells.

### 3.2. Effect of Pomiferin on Expression of ROS and NO in BV2 Cells

**Figure 3:**
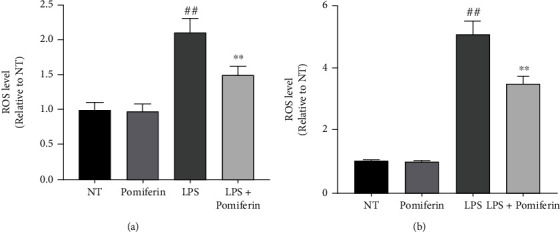
Effect of Pomiferin on expression of ROS and NO. BV2 cells were treated with LPS (100 ng/mL) and Pomiferin (1 *μ*M) for 12 h. (a) The production of ROS was detected by ROS experiment. (b) The production of NO was detected by NO experiment. Results were shown via mean ± SD (*n* = 10). ^##^ means significant difference compared with NT group. ^∗∗^ means significant difference with LPS group.

In the process of oxidative stress, ROS and NO are produced, which accumulate in large amounts and damage the surrounding tissues. In order to study the effect of Pomiferin, we treated BV2 cells with LPS (100 ng/mL) and Pomiferin (1 *μ*M) for 12 h and then detected the effect of Pomiferin on the production of ROS (Figure 3(a) and NO (Figure 3(b)) in BV2 cells. The results showed that Pomiferin inhibited the production of ROS and NO.

### 3.3. Effect of Pomiferin on Akt/Nrf2 Pathway in BV2 Cells

**Figure 4:**
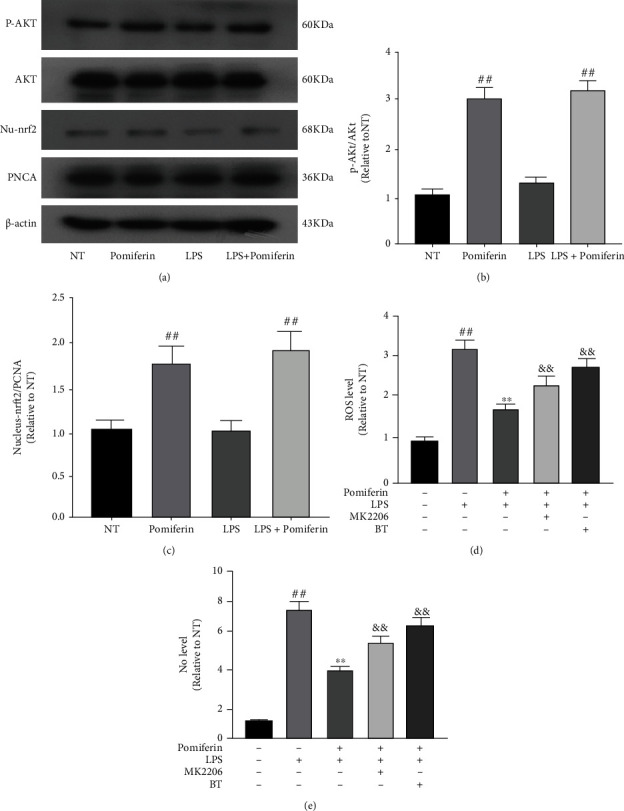
Effect of Pomiferin on Akt /Nrf2 pathway in BV2 cells. (a–c) BV2 cells were treated with LPS (100 ng/mL) and Pomiferin (1 *μ*M) for 3 h. The effect of Pomiferin on Akt and Nrf2 pathways was detected by Western blot experiment. (d, e) Then, we treated BV2 cells with MK2206 (an Akt inhibitor, 10 *μ*M) and BT (a Nrf2 inhibitor, 200 nM) for 6 h, and studied the effects of Pomiferin on ROS and NO after blocking Akt or Nrf2 pathway. Results were shown via mean ± SD (*n* = 10). ^##^ means significant difference with NT group. ^∗∗^ means significant difference with LPS group. ^&&^ means significant difference with LPS + Pomiferin group.

Akt signaling pathway is involved in basic cellular processes. Nuclear factor erythroid2-related factor 2(Nrf2) is an important protective transcription factor that generally exists in various organisms and cellules. Studies have shown that Akt/Nrf2 is involved in the regulation of oxidative stress [21, 22]. In order to investigate the mechanism of action of Pomiferin, we studied the effect of Pomiferin on Akt and Nrf2 pathways. The results showed that Pomiferin promoted the phosphorylation of Akt and the nuclear translocation of Nrf2 (Figures 4(a)–4(c)). Then, we treated BV2 cells with MK2206 (an Akt inhibitor, 10 *μ*M) and BT (a Nrf2 inhibitor, 200 nM) for 6 h and studied the effects of Pomiferin on ROS and NO after blocking Akt or Nrf2 pathway. The results showed that MK2206 or BT treatment weakened the inhibition of Pomiferin on ROS and NO (Figures 4(d) and 4(e)). These results proved that Pomiferin inhibited the production of ROS and NO through the Akt/Nrf2 pathway.

### 3.4. Effect of Pomiferin on the Expression of Proinflammatory Factors in BV2 Cells

**Figure 5:**
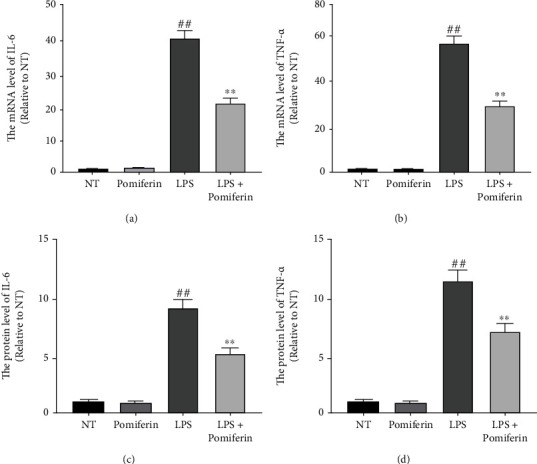
Effect of Pomiferin on the expression of proinflammatory factors in BV2 cells. BV2 cells were treated with LPS (100 ng/mL) and Pomiferin (1 *μ*M). (a, b) The mRNA levels of proinflammatory factors (IL-6 and TNF-*α*) were detected by quantitative PCR methods. (c, d) The protein levels of proinflammatory factors (IL-6 and TNF-*α*) were detected by ELISA methods. Results were shown via mean ± SD (*n* = 5). ^##^ means significant difference compared with NT group. ^∗∗^ means significant difference with LPS group.

Continued oxidative stress usually leads to an inflammatory response, and abnormal inflammatory responses produce large amounts of proinflammatory factors (IL-6 and TNF-*α*), causing damage to neurons. Therefore, we treated BV2 cells with LPS (100 ng/mL) and Pomiferin (1 *μ*M) for 12 h (mRNA) and 24 h (protein) and then detected the effect of Pomiferin on the production of proinflammatory factors (IL-6 and TNF-*α*) in BV2 cells via quantitative PCR and ELISA methods. The results showed that Pomiferin inhibited the mRNA (Figures 5(a) and 5(b)) and protein (Figures 5(c) and 5(d)) expression of proinflammatory factors (IL-6 and TNF-*α*).

### 3.5. Effect of Pomiferin on the Expression of Proinflammatory Enzymes in BV2 Cells

**Figure 6:**
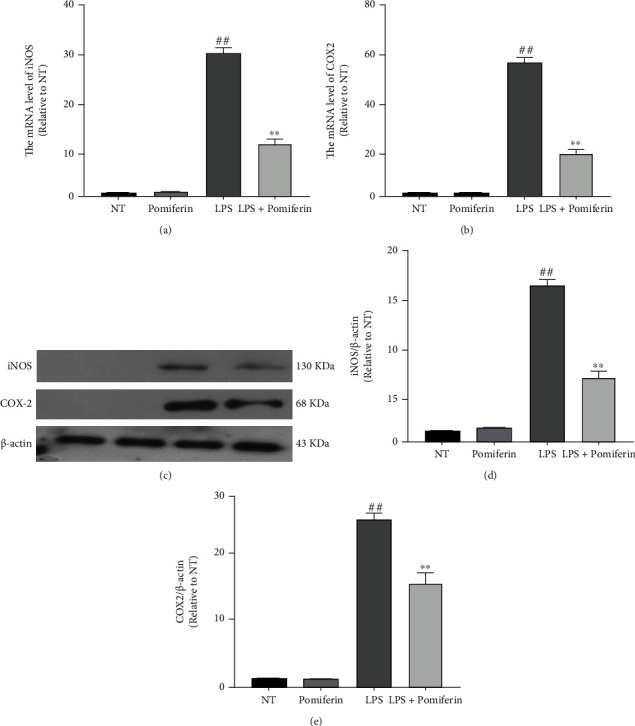
Effect of Pomiferin on the expression of proinflammatory enzymes in BV2 cells. BV2 cells were treated with LPS (100 ng/mL) and Pomiferin (1 *μ*M). (a, b) The mRNA levels of proinflammatory proteinase (iNOS and COX2) were detected by quantitative PCR methods. (c–e) The protein levels of proinflammatory proteinase (iNOS and COX2) were detected by Western blot methods. Results were shown via mean ± SD (*n* = 5). ^##^ means significant difference with NT group. ^∗∗^ means significant difference with LPS group.

In order to further study the effect of Pomiferin on inflammation, we treated BV2 cells with LPS (100 ng/mL) and Pomiferin (1 *μ*M) for 12 h (mRNA) and 24 h (protein) and then detected the effect of Pomiferin on the production of proinflammatory proteinase (iNOS and COX2) in BV2 cells via quantitative PCR and Western blot methods. The results showed that Pomiferin inhibited the mRNA (Figures 6(a) and 6(b)) and protein (Figures 5(c)–5(e)) expression of proinflammatory proteinase (iNOS and COX2).

### 3.6. Effect of Pomiferin on NF-*κ*B and MAPK Pathways in BV2 Cells

**Figure 7:**
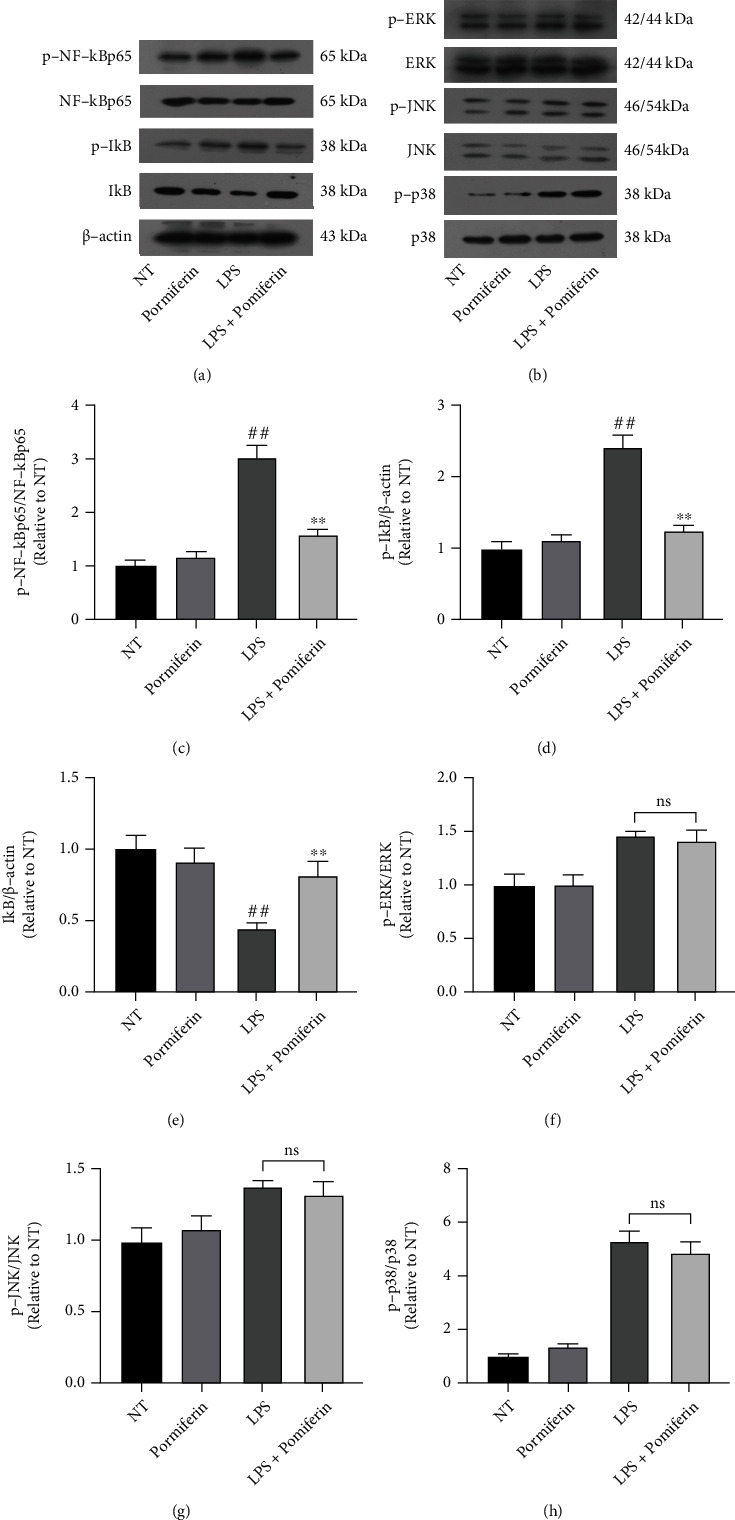
Effect of Pomiferin on NF-*κ*B and MAPK pathways in BV2 cells. BV2 cells were treated with LPS (100 ng/mL) and Pomiferin (1 *μ*M), and then, we examined the effects of Pomiferin on activation of NF-*κ*B and MAPK pathways. (a, c–e) The effect of Pomiferin on NF-*κ*B pathway-related proteins was detected by Western blot methods. (b, f–h) The effect of Pomiferin on NF-*κ*B pathway-related proteins was detected by Western blot methods. Results were shown via mean ± SD (*n* = 5). ^##^ means significant difference compared with NT group. ^∗∗^ means significant difference with LPS group.

NF-*κ*B and MAPK pathways are classical inflammatory pathways, whose activation regulates transcription of inflammatory mediators [23, 24]. To investigate the anti-inflammatory mechanism of Pomiferin, we treated BV2 cells with Pomiferin (1 *μ*M) and LPS (100 ng/mL) and then examined the effects of Pomiferin on activation of NF-*κ*B and MAPK pathways. The results showed that orange mulberry brass inhibited the activation of NF-*κ*B (Figures 7(a) and 7(c)–7(e)) and MAPK (Figures 7(b) and 7(f)–7(h)) pathways in BV2 cells.

## 4. Discussion

**Figure 8:**
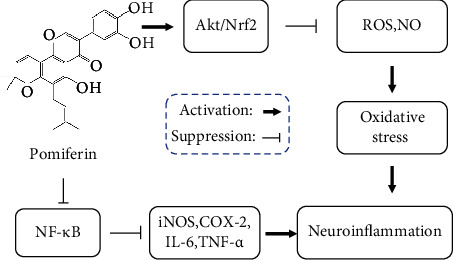
Action mechanism diagram of Pomiferin. Pomiferin exerts anti-inflammatory effects by inhibiting the production of ROS, NO, and pro-inflammatory mediators (IL-6, TNF-*α*, iNOS, and COX2) in microglia through activating Akt/Nrf2 pathway and inhibiting NF-*κ*B pathway.

In our study, we reported that Pomiferin exerts anti-inflammatory effects by inhibiting the production of ROS, NO, and proinflammatory mediators (IL-6, TNF-*α*, iNOS, and COX2) in microglia through activating Akt/Nrf2 pathway and inhibiting NF-*κ*B pathway (Figure 8).

As a flavonoid extracted from the fruit of Moraceae, Pomiferin has been reported to regulate oxidative stress [25–27]. ROS is an important indicator of oxidative stress, and it has been reported in the literature that Pomiferin regulates the release of ROS [28, 29]. In our experiment, we examined the effects of Pomiferin on the production of ROS and NO in BV2 cells. The results showed that Pomiferin inhibited the production of ROS and NO. The Akt pathway or PI3K-Akt pathway is the transduction pathway of signals corresponding to extracellular survival and growth. Activated Akt mediates downstream reactions by phosphorylation of a series of intracellular proteins that regulate cell survival, growth, proliferation, cell migration, and angiogenesis [30–32]. In our study, we examined the effect of Pomiferin on Akt phosphorylation, and the results showed that Pomiferin treatment promoted the phosphorylation of Akt. Nrf2 is a key factor in the regulation of oxidative stress, and its activation can regulate a variety of genes related to oxidative stress. Under normal circumstances, Nrf2 is located in the cytoplasm and does not play a role. After activation, Nrf2 translocates into the nucleus and binds with the antioxidant reaction elements to form the Nrf2-ARE signaling pathway, thereby regulating the expression of a series of downstream antioxidant genes and proteins and playing the antioxidant role [33–35]. In our experiment, we investigated the effect of Pomiferin on Nrf2 activation and found that Pomiferin promoted the nuclear translocation of Nrf2. Further studies showed that the inhibitors of Akt and Nrf2 could weaken the inhibition of ROS and NO by Pomiferin. It is suggested that Pomiferin inhibits the production of ROS and NO in BV2 cells by activating Akt and Nrf2 pathways.

The imbalance of oxidative and antioxidant reactions in the body will lead to oxidative stress. Continued and complex oxidative stress can trigger an inflammatory response in the body. Inflammation can lead to the imbalance of oxidation and antioxidant reactions in the body [36–38]. Therefore, regulation of oxidative stress plays an important role in inhibiting inflammatory response. The main pathological feature of neurodegenerative diseases is the degenerative loss of neurons. Inflammation plays an important role in this process. During neurodegenerative diseases, microglia are activated to mediate inflammatory responses, producing proinflammatory mediators (IL-6, TNF-*α*, iNOS, and COX2) and damaging neurons [39–41]. Therefore, the inhibition of inflammatory response is of great significance in the treatment of neurodegenerative diseases. In our experiment, we examined the effect of Pomiferin on the production of proinflammatory mediators in BV2 cells and found that Pomiferin inhibited the production of proinflammatory mediators in BV2 cells.

NF-*κ*B (enhanced *κ*-light chain of nuclear factor B) is a protein complex that plays a key role in regulating the immune response to infection. Its target genes are involved in immune response, inflammatory response, cell proliferation, antiapoptosis, etc. When the cells were stimulated, I*κ*B*α* kinase in the cytoplasm was activated, and part of I*κ*B*α* was phosphorylated, part of I*κ*B*α* was degraded by ubiquitination, and the p65 subunit associated with it was dissociated and phosphorylated [42–44]. In our experiment, we investigated the effect of Pomiferin on NF-*κ*B activation and found that Pomiferin inhibited the phosphorylation of I*κ*B*α* and p65 and the ubiquitination degradation of I*κ*B*α*. MAPK (mitogen-activated protein kinase) is a group of important transmitters that can be activated by different extracellular stimuli and make signals from the cell surface to the nucleus. It consists of ERK, JNK and P38 [45, 46]. In our experiment, we detected the effect of Pomiferin on the activation of MAPK pathway, and the results showed that Pomiferin had no effect on the activation of MAPK pathway.

## 5. Conclusion

Our study found that Pomiferin inhibited microglial inflammation by activating the Akt/Nrf2 pathway and inhibiting the NF-*κ*B pathway. In addition, the unique pharmacological properties and easy availability of Pomiferin suggest that it may be a candidate drug for antineuroinflammation. Our experiment has laid a certain foundation for further study of the effect of Pomiferin.

## Data Availability

The datasets used or analyzed during the current study are available from the corresponding author on reasonable request.

## Abbreviations

ROS: Reactive oxygen species
NO: Nitric oxide
NF-*κ*B: Enhanced *κ*-light chain of nuclear factor B
MAPK: Mitogen-activated protein kinase
TNF-*α*: Tumor necrosis factor *α*
Akt: Serine-threonine protein kinase
Nrf2: Nuclear factor erythroid-2-related factor 2
ERK: Extracellular signal-regulated kinase.

## Additional Points

*Author's Note*. A preprint has previously been published at Research Square 10.21203/rs.3.rs-718549/v1) [47].
